# Cost-Effectiveness Analysis of Switching from Trivalent to Quadrivalent Seasonal Influenza Vaccine in Argentina

**DOI:** 10.3390/vaccines9040335

**Published:** 2021-04-01

**Authors:** Analia Urueña, Paula Micone, Cecilia Magneres, Joaquin Mould-Quevedo, Norberto Giglio

**Affiliations:** 1 Centro de Estudios para la Prevención y Control de Enfermedades Transmisibles, Universidad Isalud, Buenos Aires C1095AAS, Argentina; uruenaanalia@gmail.com; 2 Hospital Carlos G Durand, Buenos Aires 1405, Argentina; paulamicone@gmail.com; 3 Seqirus S.A., Buenos Aires B1636AKJ, Argentina; 4 Seqirus USA Inc., Summit, NJ 07901, USA; Joaquin.Mould-Quevedo@seqirus.com; 5 Hospital de Niños Ricardo Gutierrez, Buenos Aires 1425, Argentina; norbergiglio@gmail.com

**Keywords:** influenza, vaccine, cost-effectiveness, trivalent, quadrivalent, Argentina, TIV, QIV

## Abstract

The burden of seasonal influenza disease in Argentina is considerable. The cost-effectiveness of trivalent (TIV) versus quadrivalent influenza vaccine (QIV) in Argentina was assessed. An age-stratified, static, decision-tree model compared the costs and benefits of vaccination for an average influenza season. Main outcomes included: numbers of influenza cases; general practitioner (GP) visits; complicated ambulatory cases; hospitalizations; deaths averted; and costs per quality-adjusted life years (QALYs) gained. Epidemiological data from Argentina for 2014–2019 were used to determine the proportion of A and B strain cases, and the frequency of mismatch between vaccine and circulating B strains. To manage uncertainty, one-way and probabilistic sensitivity analyses were performed. Switching from TIV to QIV would prevent 19,128 influenza cases, 16,164 GP visits, 2440 complicated ambulatory cases, 524 hospitalizations, and 82 deaths. Incremental cost–effectiveness ratios (ICERs) per QALY were 13,590 and 11,678 USD from the payer’s and societal perspectives, respectively. The greatest health benefits and direct medical cost savings would occur in ≥ 65-year-olds. One-way sensitivity analyses demonstrated the principal drivers of ICER to be vaccine acquisition costs, environmental B strain predominance, and B strain mismatch. Introducing QIV in Argentina would be beneficial and cost-effective relative to TIV, particularly in older adults.

## 1. Introduction

Seasonal influenza is a major cause of morbidity and mortality worldwide, affecting individuals of all ages. There are four known strains of influenza virus, A, B, C, and D. Seasonal influenza disease in humans results from A and B strain infections, with A strain viruses being responsible for the vast majority of influenza cases [1]. The burden of seasonal influenza disease and influenza-related complications in Argentina is considerable, with numbers of cases peaking between the months of May and October each year [2].

Vaccination is currently considered to be the most effective public health measure against seasonal and pandemic influenza [1,3]. Since 2011, the annual receipt of trivalent influenza vaccine (TIV) containing antigens derived from two A strains and one B strain has been recommended in the Argentine National Immunization Program for: (1) children 6 months to 2 years of age; (2) adults ≥ 65 years of age; (3) healthcare workers; (4) pregnant women; and (5) other groups considered to be at high risk of influenza-related complications, such as the immunocompromised, and those with diabetes, respiratory, cardiovascular, and other conditions [4].

In the 1980s, two distinct lineages of B strain virus emerged which continue to co-circulate today in both the northern and southern hemispheres, B/Victoria and B/Yamagata [5]. Consequently, the development of quadrivalent influenza vaccines (QIVs) containing antigens derived from two A strains and the two B strains has been of significant benefit, with these vaccines offering additional protection compared with TIVs in terms of B strain mismatch [6]. As reported by Palekar et al., influenza viruses of both B/Yamagata and B/Victoria lineage circulate each year in Latin American and Caribbean countries, with 4500–7000 cases of B strain disease reported within the region annually during 2010–2017 [7]. Jamotte et al. estimate that the use of QIV in Brazil, Colombia, and Panama would significantly reduce the burden of seasonal influenza disease in these countries [8]. Studies from Mexico and Brazil have shown that the use of quadrivalent rather than trivalent vaccines in these countries would be cost-effective [9,10,11]. These and other data suggest that the introduction of QIV in Argentina could potentially be of benefit.

The present study was performed to evaluate the impact and cost-effectiveness of switching from the current TIV immunization policy in Argentina, to use of QIV in all groups.

## 2. Materials and Methods

**Figure 1:**
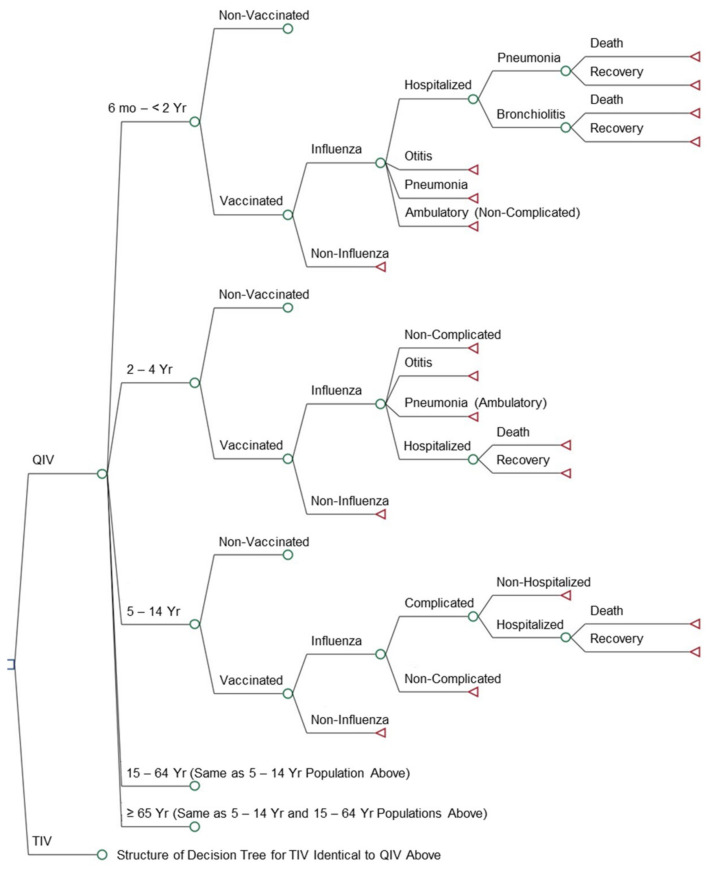
Static decision tree model used to compare the cost-effectiveness of quadrivalent influenza vaccine (QIV) with trivalent influenza vaccine (TIV).

*Model Structure*: A static decision-tree model was developed using TreeAge Pro 2020 R1.2 software (TreeAge Software LLC., Williamstown, MA, USA) to compare the clinical and economic benefits of QIV (Afluria^®^ Quadrivalent; Seqirus Pty Ltd., Melbourne, Australia) with TIV (representing the current universal vaccination strategy in Argentina) in terms of costs and quality-adjusted life years (QALYs; Figure 1).

The model predicted annual outcomes nationally including numbers of influenza cases; general practitioner (GP) visits; complicated ambulatory cases of acute otitis media or pneumonia; hospitalizations; deaths; and costs per QALYs gained. Costs considered in the model included the management of influenza disease and influenza-related complications. Direct and indirect costs were assessed from the payer’s and societal perspectives, respectively. The following age groups were analyzed: (1) 6 months– < 2 years of age; (2) 2–4 years (with risk factors); (3) 5–14 years (with risk factors); (4) 15–64 years (with risk factors); and (5) ≥ 65 years of age. For the 6 month– < 2-year-old population, rates of resource use for outpatient pneumonia, acute otitis media, and hospitalizations for pneumonia and bronchiolitis were considered [12,13,14,15,16]. For children 2–4 years of age with risk factors, quantified events were cases of non-complicated outpatient influenza, acute otitis media, and outpatient and hospitalized pneumonia. For the 5–64-year-old age group with risk factors, events were non-complicated and complicated influenza with hospitalizations.

The time horizon analyzed for QIV and TIV strategies was one year for both costs and events. For the QALYs estimate, life expectancy was considered for each age group according to 2018 vital statistics data [17]. An estimate discount of 3% per year was applied for years gained from premature deaths prevented. Incremental cost-effectiveness ratio (ICER) results of the analysis were expressed as the incremental overall cost difference of QIV vaccination versus TIV divided by the difference of the total QALYs gained for each vaccine. In all cases, local data from Argentina were prioritized for use. If Argentine data were not available, data from other countries were obtained from international, peer-reviewed, scientific literature.

**Figure 2:**
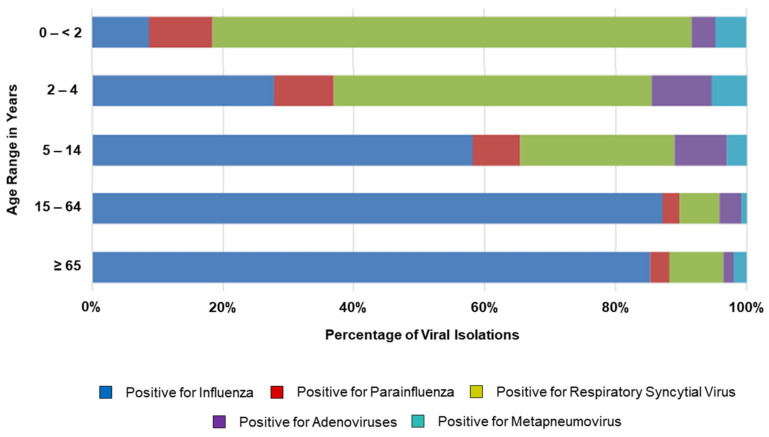
Proportions of viral isolations by age group in Argentina 2014–2019 [19].

**Figure 3:**
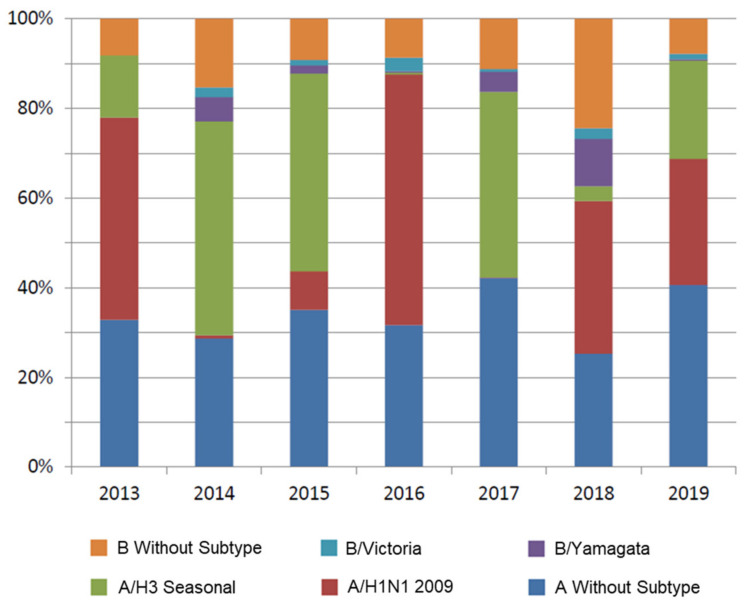
Influenza cases in Argentina 2013–2019 by subtype and unclassified [19].

*Model Inputs*: The main input data and model assumptions are presented in Table 1 and Table 2, Figure 2 and Figure 3.

*Population Data and Proportions of People with Risk Factors*: The estimated target populations for vaccination in the 6 month–< 2-year-old and ≥ 65-year-old groups were based on data from the most recent National Census of Population and Housing conducted in 2010 [18]. Estimates from the National Immunization Program and the National Risk Factor Survey were considered to calculate the proportions of individuals with risk factors for influenza-associated complications in the 2–4, 5–14, and 15–64-year-old age groups, which were assumed to be 11.5% of the total population in each age group [27]. Levels of vaccination coverage for each age group were obtained from the National Immunization Program [28], similar levels of vaccination coverage were assumed for both TIV and QIV (Table 1).

**Figure 4:**
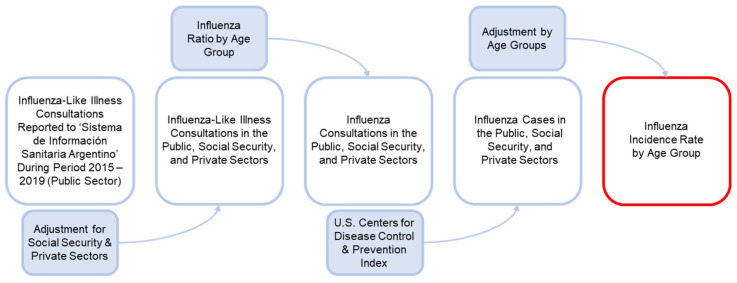
Process to estimate influenza incidence rate by age group.

*Influenza Attack Rate*: The average incidence rate for influenza in each age group was estimated based on cases of influenza-like illness (ILI) and viral isolates reported to the national surveillance system “Sistema Integrado de Información Sanitaria Argentino” (SISA) by age group for period 2015–2019 [29] (Figure 4).

There are three healthcare sectors in Argentina: (1) the public sector, which provides care to those with no health insurance; (2) the social security sector, which provides care to those who have state health insurance through trade unions; and (3) the private sector, which provides care for those with pre-paid health insurance policies. As the SISA largely collects information from the public healthcare sector, the incidence rates of influenza were adjusted to include ILI cases occurring within the social security and private sectors, as described previously by Nguyen et al. [15]. As previously reported [15], the model also assumed that in the ≥ 65-year-old age group—whose healthcare cover is mainly provided by the social security program for retirees and pensioners “Programa de Atención Médica Integral” (PAMI)—40% of consultations were conducted with public rather than private sector effectors.

To estimate the real incidence rate of influenza, the model considered influenza cases that would not be registered by the SISA because individuals had not attended consultations; for this, the underreporting index used by the U.S. Centers for Disease Control and Prevention (CDC) was used to estimate the burden of seasonal influenza disease according to age group [30]. However, these average, age group-specific incidence rates were estimated in the context of partial TIV population coverage, given that the influenza vaccination program was first implemented in Argentina in 2011. Thus, these rates include vaccinated and non-vaccinated individuals, and are also influenced by levels of vaccine efficacy and vaccination coverage. Therefore, in order to estimate an influenza incidence rate in the non-vaccinated population, the following formulas were used in the model: non-vaccinated incidence (In-v) = Itiv/((1-PRF) + ((1-VC) + (1-EfTIV)*VC)* PRF); where: Itiv = current incidence (vaccination with TIV); VC = vaccination coverage; PRF = proportion of the population with risk factors; EfTIV = efficacy of TIV; and EfQIV = efficacy of QIV. In the case of the 6 month– < 2-year-old and ≥ 65-year-old age groups, PRF was not applied because the entire cohort was considered to be a population “at risk”; therefore, the applied formula was: non-vaccinated incidence (In-v) = Itiv/((1-VC) + VC*(1-EfTIV)). Thus, incidence rates of influenza without vaccination were estimated for each age group and were applied to the model (Table 1).

*Circulation of Influenza Viruses*: Although influenza may circulate throughout the entire year in Argentina, the influenza season typically occurs from March to September, with infection rates peaking between May and August. Data on the proportions of influenza virus isolates by age group, the proportions of A and B strain viruses circulating each season, and the percentages of B/Yamagata versus B/Victoria strain viruses observed during 2014–2019 were obtained from the National Ministry of Health (National Epidemiology Department) and the World Health Organization (WHO) [2,19] (Table 1). Vaccine B strain mismatch was assessed by comparing information on circulating B strains with the WHO-recommended composition of TIV for use in the southern hemisphere [26]. To ensure consistency and validity, average values for the entire study period (2013–2019) were calculated for B strain circulation (18%) and B strain match (66%; Table 1), these values were applied to all age groups.

*Vaccine Efficacy*: Currently, no head-to-head clinical trials have been performed to compare the efficacy of TIV against QIV. Age-specific vaccine efficacy values for TIV and QIV were taken from Tricco et al. [20] and considered similar levels of efficacy against A strain viruses for both TIV and QIV (58–61%; Table 1). The efficacy of QIV was considered to be similar against both B/Yamagata and B/Victoria, and similar to the efficacy of TIV against matched B strains (66–77%). Thus, estimated QIV incremental vaccine efficacy is directly affected by B strain circulation and mismatch, and may vary from season to season. In our base case model and considering the 2014–2019 period with an average B strain mismatch of 34.24%, average age-specific QIV incremental vaccine efficacy was 4.10–4.79%. However, if we considered the 2016 season with B strain mismatch of 8.30%, and the 2017 season with B strain mismatch of 87.50%, estimated QIV incremental vaccine efficacies were 0.66–0.77% and 9.24–10.78%, respectively. In addition, studies have described TIV as providing cross-protection against the B lineage not included in vaccines at 68% of the efficacy observed against the included B lineage; however, other randomized clinical trials report either insignificant or low levels of cross-protection [31,32]. Therefore, given the lack of certainty around this issue, the base scenario of our analysis did not consider cross-protection. The cross-protection variable was only included in the sensitivity analysis.

*Use of Resources and Costs*: Estimates of resource use for ILI in children, adults, and older adults were taken from scientific publications reporting data from Argentine studies (Table 1) [12,15,21,33]. In the absence of local/Argentine data to estimate the proportions of influenza cases that may become complicated, require hospitalization, or be fatal as a result of complications, working values were taken from international, peer-reviewed, scientific literature [13,14,15,16]. The direct costs of treating influenza considered the costs of hospitalization, GP visits, laboratory testing, and radiologic images, and were obtained from previous reports (Table 1) [12,15,21]. In all cases, values were updated according to the price inflation index of the Argentine healthcare system with an exchange rate of 1.000 U.S. dollar (USD) = 61.350 Argentine pesos (ARS). Given that unit costs were taken from local tariffs, which are known to underestimate real values, a cost increase was applied according to the Moscoso and Clark index for those under 65 years of age (those ≥65 had already been adjusted), as in our previous study [15,34]. Following the human capita approach, indirect costs applied for only the 18–64-year-old group, and were estimated considering the average monthly take-home pay of employees in 2019, adjusted by the unemployment rate in 2019 using data from the Ministry of Labor, Employment and Social Security, and the National Institute of Statistics and Censuses (INDEC) [22,23].

**Figure 5:**
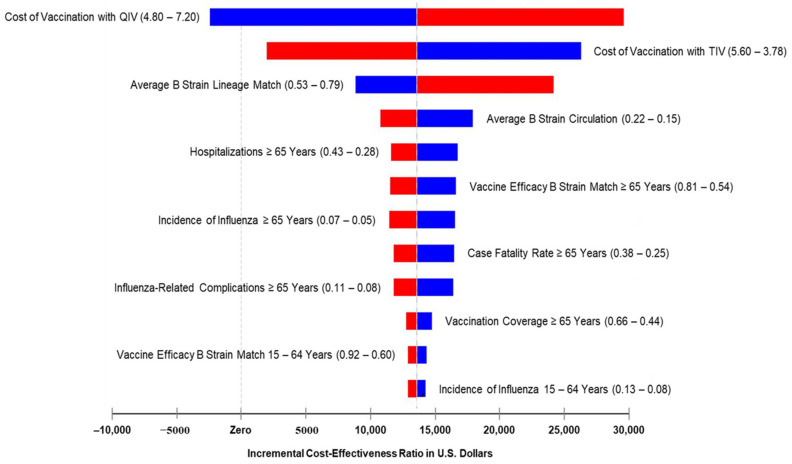
Deterministic sensitivity analysis, tornado diagram. Incremental cost-effectiveness of QIV versus TIV.

The price per dose of adult QIV was obtained from the Pan American Health Organization (PAHO) revolving fund for 2020 influenza vaccines [24]. Prices per dose of pediatric and adult TIV and QIV (Afluria Quadrivalent) formulations were based on values obtained from the manufacturer and PAHO [24,25]. To calculate QALYs, we used the Spanish utility findings previously published by Garcia et al. [35]. To manage uncertainty, univariate and probabilistic sensitivity analyses were performed on the variables considered to have the highest impact: rates of complicated influenza; case fatality ratios; proportions of B strain circulation and B strain mismatch; vaccine efficacy against B strains; vaccination coverage; the price of vaccines; and influenza-related direct costs. As part of our univariate analyses, a deterministic tornado-type sensitivity analysis was performed using a ± 20% value for each variable following a triangular distribution (Figure 5).

Lastly, we constructed (1) a more favorable scenario with high B strain mismatch (at 87%) because this was reported for the 2017 season and could be repeated, and (2) another less favorable scenario considering TIV-induced cross-protection at a level described by Tricco et al. [20].

## 3. Results

### 3.1. Estimated Healthcare Outcomes

Analysis of health outcomes from the payer’s perspective demonstrated that switching from use of TIV to QIV would prevent an estimated 19,128 cases of influenza, 16,164 influenza-associated GP visits, 2440 complicated ambulatory cases, 524 hospitalizations, and 82 influenza-associated deaths in total across all age groups (Table 3).

The greatest numbers of events avoided would occur in the “at risk” 15–64-year-old and ≥ 65-year-old populations, with 8027 and 6966 cases of influenza, 7027 and 6313 GP visits, 843 and 417 complicated ambulatory cases, 157 and 236 hospitalizations, and 6 and 75 influenza-associated deaths averted in these two age groups, respectively (Table 3). Cases of influenza were predicted to total 358,850, 183,835, and 164,707 if the population were non-vaccinated (data not shown), TIV-vaccinated, or QIV-vaccinated; influenza-associated hospitalizations were predicted to be 9496, 5138, and 4614 cases; and 1591, 855, and 773 influenza-associated fatalities were predicted, respectively (Table 3). Overall, both TIV and QIV would significantly reduce the incidence of all health outcomes analyzed, with QIV being the more effective of the two vaccines in each scenario. Therefore, compared with TIV, immunization with QIV would reduce total numbers of influenza cases by 10.4% (164,707/183,835), GP visits by 10.4% (138,710/154,874), complicated ambulatory cases by 10.2% (21,380/23,820), hospitalizations by 10.2% (4614/5138), and deaths by 9.6% (773/855).

### 3.2. Incremental Cost-Effectiveness Ratios

The ICER per QALY was 13,590 USD from the payer’s perspective, and 11,678 USD from the societal perspective. These values are considerably below the cost-effectiveness threshold proposed by the WHO of three times gross domestic product (GDP) per capita in Argentina (i.e., ~30,000 USD), and close to the 2019 GDP per capita of 10,006 USD [36,37], suggesting that the introduction of QIV would be highly cost-effective.

### 3.3. Sensitivity and Scenario Analysis

**Figure 6:**
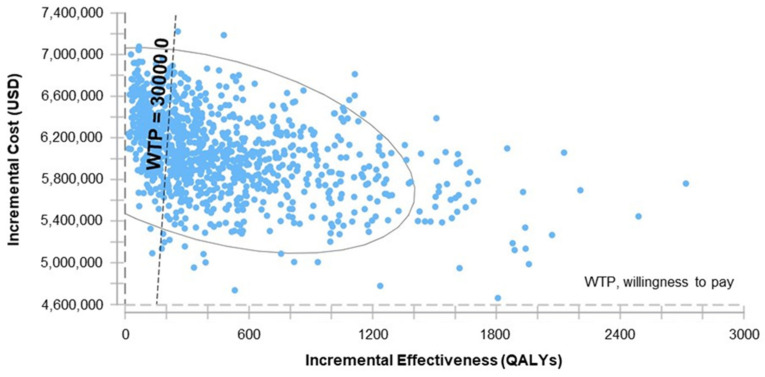
Probabilistic sensitivity analysis. Monte Carlo Simulations (*N* = 10,000). Incremental cost-effectiveness of QIV versus TIV.

**Figure 7:**
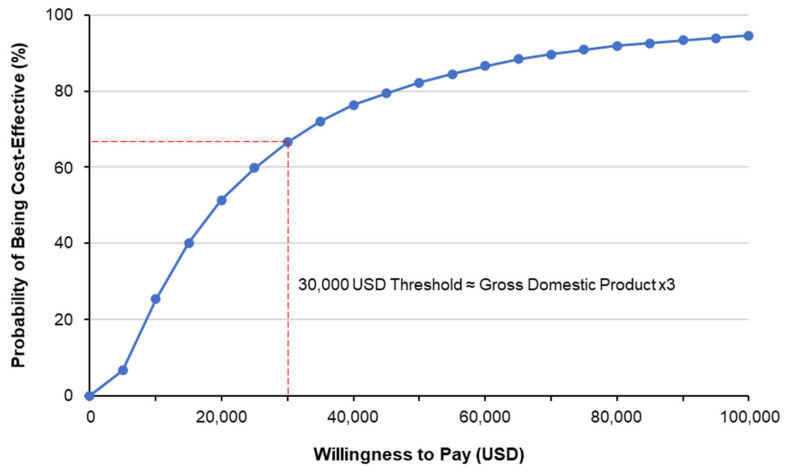
Cost-effectiveness acceptability curve.

One-way sensitivity analyses demonstrated the principal drivers of ICER to be the acquisition costs of TIV and QIV, degree of environmental B strain dominance, and the frequency of B strain mismatch (Figure 5). Lesser, but still significant, drivers of ICER were: hospitalizations, vaccine efficacy against matched B strains, the incidence of influenza, case fatality rates, influenza-related complications, and levels of vaccination coverage, all in the ≥ 65-year-old population; vaccine efficacy against matched B strains and the incidence of influenza in the 15–64-year-old population; and cases of ambulatory pneumonia in under 2-year-olds (Figure 5). Probabilistic sensitivity analysis (Figure 6) and the cost-effectiveness acceptability curve presented in Figure 7 show that, in 63% of simulations and with a willingness to pay of ~3 times GDP per capita (30,000 USD, as recommended by the WHO), vaccination with QIV would be cost-effective relative to TIV.

The ICER per QALY gained in the high B strain mismatch scenario (using 87% mismatch, as in the 2017 season) was 3245 USD; with TIV-induced cross-protection against the absent B strain at 66.5%, the ICER per QALY gained was 47,023 USD.

## 4. Discussion

Studies from all regions of the world have demonstrated the significant economic benefits of QIV use compared with TIV [9,10,11,16,38,39,40,41,42,43,44,45,46]. Currently in Argentina, the National Immunization Program recommends annual receipt of TIV for individuals classified as “at risk”. While the TIV immunization strategy has largely been successful, correctly predicting which of the two possible B strain lineages should be included in the TIV each season is an ongoing challenge in Argentina, as it is in the rest of the world. Therefore, the introduction of QIV offers the benefit of guaranteed coverage for both B/Yamagata and B/Victoria strains each year. To date, no peer-reviewed scientific papers have been published reporting analyses of QIV cost-effectiveness in Argentina. Given the absence of data in this area, the usefulness of such analysis, and the benefits of such a study in providing information to public health policy makers, we evaluated the cost-effectiveness of QIV in Argentina compared with the current TIV immunization strategy. Briefly, QIV was estimated to be cost-effective relative to TIV, preventing 19,128 cases of influenza, 16,164 influenza-associated GP visits, 2440 complicated ambulatory cases, 524 hospitalizations, and 82 influenza-related deaths annually.

The primary benefit of introducing QIV is the avoidance of mismatch between the predominant B strain circulating in the environment and the B strain included in the TIV formulation for a given season. A recent study by Palekar et al. compared the B strains included in TIVs with environmental B strain predominance in Latin America and the Caribbean between 2010–2017 and found B strain mismatch to have occurred in 32% of seasons assessed [7]. When defining seasons of co-dominance between the B lineages as mismatch seasons, the rate of B strain mismatch during 2010–2017 increased to 52% of seasons [7]. These and other data demonstrate B strain mismatch to be a common occurrence across Latin American countries, including Argentina, and consequently indicate a need for QIV and the potential for improved public health as a result of its use.

While the data presented in this report estimates that switching from TIV to QIV would result in significant benefits in terms of both health outcomes and cost savings, it should be considered that these benefits would be greatly affected by vaccine costs, primarily, and the degree of mismatch and B strain predominance from season to season. The cost-effectiveness of QIV would be higher in seasons of high B strain predominance and mismatch, and lower in seasons of low-level B strain circulation and mismatch. These benefits would be markedly reduced when TIV-induced cross-protection against the mismatched B strain is considered; this is a field where information is still scarce and very dissimilar, but which could undoubtedly impact cost-effectiveness. A further consideration regarding factors which would affect the extent of QIV-associated benefits is rates of vaccination coverage in Argentina, particularly in the adult population, where rates usually do not exceed 50%.

Exceptionally high influenza vaccination coverage was achieved in 2020 in the adult population, probably due to the emergence of severe acute respiratory syndrome coronavirus 2 (SARS-CoV-2) and the fact that the elderly population perceived a greater risk of becoming ill. Although during the first pandemic year the circulation of influenza viruses was very low in Argentina and other regional countries, the co-circulation of both viruses could occur thereafter, demanding strategies to manage the burden of disease from these two pathogens in parallel [47]. Co-infection with influenza and SARS-CoV-2 is known to occur [48,49,50,51]. In addition, influenza and SARS-CoV-2 infections are both most severe in the same high-risk populations—older adults, the immunocompromised, and those with pre-existing cardiovascular and respiratory conditions. Thus, optimal measures are required to minimize the impact of influenza in order to spare the essential resources needed to effectively manage the COVID-19 pandemic. Given that co-circulation and co-infection are likely to occur in the future, it is reasonable to suggest that combined or concomitant administration of seasonal influenza and SARS-CoV-2 vaccines to high-risk/at-risk populations may be a logical and beneficial public health strategy. However, it should be noted that, at the time of this publication, given the lack of safety and efficacy data on COVID-19 vaccines being administered simultaneously with other vaccines, co-administration is not currently recommended [52].

The first COVID-19 case in Argentina was reported in March 2020, having been imported from Italy [53]. Although there was no evidence that SARS-CoV-2 had circulated in Argentina before 2020, there was some evidence that the novel virus was circulating in Brazil in November 2019 [54]. Taking into consideration that during 2019 there could have been cases of COVID-19 in Argentina not yet detected, and that these cases could have impacted on our results, an ad hoc analysis was carried out excluding the year 2019; under this potential scenario the ICER was 10,835.26 USD per QALY, concluding no significant impact related to influenza-like illness and mismatch. This observation was consistent with previous years of seasonal influenza.

Limitations to this study include the use of international evidence rather than Argentine data to estimate some model parameters, including rates of hospitalized cases and complicated ambulatory cases. Another limitation is that real incidence rates for a non-vaccinated population are simply unobtainable in Argentina, because almost a decade has passed since the start of the influenza vaccination program. This obstacle was overcome by other researchers by the inclusion of the incidence of randomized clinical trials where vaccine efficacy was originally tested in their models [10,55,56,57]. However, these trials have been conducted in other countries, many of them in the northern hemisphere, where climate issues and population behavior may affect influenza attack rates in a very different way, and hence may not be representative of local epidemiology. Thus, we preferred to estimate the non-vaccinated population incidence based on local information taking into consideration the current incidence rate of influenza among TIV recipients, rates of vaccination coverage, and TIV efficacy. In addition, estimated incidence rates were based mainly on public sector data and assumed a degree of underreporting, real incidence rates are therefore likely to be higher than those predicted. Finally, although in Argentina the use of antiviral medication among hospital outpatients and in the private sector is generally low, it should be noted that antiviral use was not taken into consideration in our model.

The results of this study indicate that switching from use of TIV to QIV would be a cost-effective public health measure in Argentina, resulting in a reduction in the burden of seasonal influenza-related morbidity and mortality, particularly in seasons of high B strain circulation and mismatch.

## Figures and Tables

**Table 1:** Main model inputs.

	6 Mo–< 2 Yr	2–4 Yr	5–14 Yr	15–64 Yr	≥ 65 Yr
Population at Risk (N) [18]	1,044,147	258,670	826,015	3,248,720	4,863,479
Incidence Rate of Influenza Per 100 People (%)	5.7	7.5	8.6	11	6.3
Complicated Cases (%)	43	25	18	13	9.4
Hospitalizations (%)	3.4	3.4	16	16	36
Case Fatality Rate (%)	0.2	1.7	1.7	4.0	32
Average B Strain Circulation 2013–2019 (%) [2,19]	18	18	18	18	18
Average B Strain Match 2013–2019 (%) [2,19]	66	66	66	66	66
TIV and QIV Efficacy Against A Strains (%) [8,20]	59	59	59	61	58
TIV Efficacy Against Matched B Strains (%) [8,20]	66	66	77	76	68
TIV Efficacy Against Mismatched B Strains (%) [8,20]	43	43	51	50	45
QIV Efficacy Against B Strains (%) [8,20]	66	66	77	76	68
Vaccination Coverage (%) [18]	75	83	50	50	55
Cost of Ambulatory Non-Complicated Case (USD) [15]	16.12	16.12	16.12	42.10	42.10
Cost of Ambulatory Complicated Case (USD) [12,15,21]	53.50	53.50	53.50	42.10	42.10
Cost of Hospitalized Case (USD) [12,15,21]	1545.47	1545.47	1600.09	1600.09	1600.09
Indirect Ambulatory Costs [22,23]	–	–	–	100	–
Indirect Hospitalization Costs [22,23]	–	–	–	200	–
Cost of TIV per Dose (USD) [24,25]	3.30	4.00	4.70	4.70	4.70
Cost of QIV per Dose (USD) [24,25]	4.20	5.10	6.00	6.00	6.00

Mo, months of age; Yr, years of age; TIV, trivalent influenza vaccine; QIV, quadrivalent influenza vaccine; USD, U.S. dollars.

**Table 2:** Percentage B strain mismatch 2014–2019 [2,26].

	WHO-Recommended Trivalent Vaccine Composition				Proportion of All Environmental Circulating Strains						B Strain Mismatch
	A/H1N1	A/H3N2	B/Yama	B/Vic	A/H1N1	A/H3N2	B/Yama	B/Vic	Total B Strains	Vaccine B Strains	
2014	California/ 7/09	Texas/ 50/12	Massachusetts/ 2/12	N/A	1.0%	76.0%	16.0%	6.0%	22.0%	72.7%	27.3%
2015	California/ 7/09	Switzerland/ 9715293/13	Phuket/ 3073/13	N/A	14.0%	74.0%	7.0%	5.0%	12.0%	58.3%	41.7%
2016	California/ 7/09	Hong Kong/ 4801/14	N/A	Brisbane/ 60/08	87.0%	1.0%	1.0%	11.0%	12.0%	91.7%	8.3%
2017	Michigan/ 45/15	Hong Kong/ 4801/14	N/A	Brisbane/ 60/08	0%	83.0%	14.0%	2.0%	16.0%	12.5%	87.5%
2018	Michigan/ 45/15	Singapore/ INF/16 *	Phuket/ 3073/13	N/A	57.0%	5.0%	31.0%	7.0%	38.0%	81.6%	18.4%
2019	Michigan/ 45/15	Switzerland/ 8060/17	N/A	Colorado/ 06/17	51.0%	40.0%	2.0%	7.0%	9.0%	77.8%	22.2%
Average					35.0%	46.5%	11.8%	6.3%	18.2%	65.8%	34.2%

B/Yama, B/Yamagata lineage; B/Vic, B/Victoria lineage; * Singapore/INFIMH-16-0019/16.

**Table 3:** Estimated healthcare outcomes in TIV versus QIV recipients.

	Total Influenza Cases	General Practitioner Visits	Complicated Ambulatory Cases			Hospitalizations			Deaths
			AOM	Pneumonia		Pneumonia		Bronchiolitis	
**Trivalent Influenza Vaccine**									
6 Mo–< 2 Yr	18,920	10,186	7511	586		400		235	0
2–4 Yr	7078	5092	1656	92		237		–	4
5–14 Yr	15,063	12,352	2283			428		–	7
15–64 Yr	69,628	60,959	7301			1368		–	54
≥ 65 Yr	73,146	66,285	4391			2470		–	790
Total	183,835	154,874	23,820			5138			855
Quadrivalent Influenza Vaccine									
6 Mo–< 2 Yr	17,144	9230	6806		531	362		213	0
2–4 Yr	6415	4615	1501		83	215		–	4
5–14 Yr	13,367	10,961	2027			379		–	6
15–64 Yr	61,601	53,932	6458			1211		–	48
≥ 65 Yr	66,180	59,972	3974			2234		–	715
Total	164,707	138,710	21,380			4614			773
Events Avoided									
6 Mo–< 2 Yr	1776	956	705				38		0
2–4 Yr	663	477	155				22		0
5–14 Yr	1696	1391	256				49		1
15–64 Yr	8027	7027	843				157		6
≥ 65 Yr	6966	6313	417				236		75
Total	19,128	16,164	2440				524		82

AOM, acute otitis media; Mo, months of age; Yr, years of age.

## Data Availability

The data presented in this study are available on request from the corresponding author.
